# Mechanisms Associated with Activation of Intracellular Metabotropic Glutamate Receptor, mGluR5

**DOI:** 10.1007/s11064-016-2026-6

**Published:** 2016-08-11

**Authors:** Yuh-Jiin I. Jong, Karen L. O’Malley

**Affiliations:** Department of Neuroscience, Washington University School of Medicine, 660 South Euclid Ave, Saint Louis, MO 63110 USA

**Keywords:** Metabotropic, Glutamate, GPCR, Calcium

## Abstract

The group 1 metabotropic glutamate receptor, mGluR5, is found on the cell surface as well as on intracellular membranes where it can mediate both overlapping and unique signaling effects. Previously we have shown that glutamate activates intracellular mGluR5 by entry through sodium-dependent transporters and/or cystine glutamate exchangers. Calibrated antibody labelling suggests that the glutamate concentration within neurons is quite high (~10 mM) raising the question as to whether intracellular mGluR5 is maximally activated at all times or whether a different ligand might be responsible for receptor activation. To address this issue, we used cellular, optical and molecular techniques to show that intracellular glutamate is largely sequestered in mitochondria; that the glutamate concentration necessary to activate intracellular mGluR5 is about ten-fold higher than what is necessary to activate cell surface mGluR5; and uncaging caged glutamate within neurons can directly activate the receptor. Thus these studies further the concept that glutamate itself serves as the ligand for intracellular mGluR5.

## Introduction

Signal transduction from G protein coupled receptors (GPCRs) has traditionally been thought to emanate from the cell surface where many signaling complexes are clustered and where extracellular stimuli can interact with GPCR ligand binding domains. Recently, however, numerous GPCRs have also been found to be associated with various intracellular membranes where, in certain cases, they activate intracellular signaling machinery leading to unique functional responses [1–11]. One such receptor is the metabotropic glutamate receptor, mGluR5, which is highly expressed on intracellular membranes including the ER and nuclear membranes throughout the CNS [12, 13]. Endogenous nuclear mGluR5 couples to G_q_ and PLC to generate IP_3_-mediated Ca^2+^ release within the nucleus and activation of intracellular mGluR5 generates unique Ca^2+^ responses as well as downstream signaling cascades distinct from cell surface counterparts, [14, 15]. These observations and those by others challenge the notion that cells only interact with their environment at the plasma membrane to bring about long term changes.

The question arises then as to what ligand activates intracellular mGluR5 and mechanistically how activation is achieved. The most parsimonious answer is that as the natural ligand, glutamate itself may activate intracellular mGluR5. Glutamate uptake is mediated by at least five sodium-dependent transporter proteins that are present on glial and neuronal cells as well as the chloride-dependent cystine-glutamate exchanger [16–21]. Previous data show that both of these uptake systems are responsible for transporting glutamate into striatal, hippocampal and/or spinal cord dorsal horn neurons to activate mGluR5 [13, 22–24]. Conditions that block the transporters (i.e., chloride-free buffers and the compound l-cystine for the cystine/glutamate exchanger; sodium free buffers and the compound, threo-β-benzyloxyaspartate for sodium-dependent excitatory amino acid transporters) reduce agonist uptake in mGluR5-expressing neurons [13, 22–24]. Moreover, uptake of radiolabeled quisqualate, an mGluR5 agonist, and glutamate is also observed in isolated nuclei, which can be blocked with chloride-free buffers or by applying the same transporter blockers. Thus, 90–95 % of all ligand-induced intracellular responses can be accounted for by these ligand transport processes [13, 22–24].

Besides mechanisms by which glutamate can enter the cell, another limitation to the notion that endogenous ligand can activate intracellular mGluR5 is the idea that cytoplasmic glutamate concentrations are in the mM range. Indeed, 10 mM is frequently used as the concentration of cytoplasmic glutamate with levels ranging up to 100–200 mM within vesicles [25]. If cytoplasmic glutamate concentrations are indeed 10 mM then an intracellular glutamate receptor would be maximally activated or possibly desensitized long before a new bolus of glutamate entered the cell. To address these issues we have used cellular, optical and molecular techniques to determine the (1) intracellular localization of glutamate; (2) concentrations necessary to activate cell surface and intracellular mGluR5; and (3) the effects of uncaging caged glutamate within neurons.

## Materials and Methods

### Cell Culture and Transfection

Primary cultures of striatal neurons were prepared from postnatal day 1 rat pups as previously described [13]. The cells were plated onto 12-mm poly-d-lysine-coated glass coverslips (60,000/coverslip) for immunostaining or Ca^2+^ imaging. Cells were cultured in humidified air with 5 % CO_2_ at 37 °C for 11–15 days before use. For experiments using the microplate reader, cultures were plated at 40,000 cells per well in black-walled, clear-bottomed 96-well plates and then cultured as above. Striatal cultures were transfected with plasmid mito-eYFP (gift from Dr. Ian Reynolds; Department of Pharmacology, University of Pittsburgh, Pittsburgh, Pennsylvania) using lipofectamine 2000 (Invitrogen, Carlsbad, CA) on DIV 9 and then immunostained on DIV 10.

### Immunocytochemistry

Striatal cultures were fixed, blocked, and incubated as described [13]. Primary antibodies include mouse anti-glutamate (1:5000; ImmunoStar, Inc., Hudson, WI) and goat anti-HSP60 (1:100; Santa Cruz Biotechnology, Santa Cruz, CA). Secondary antibodies include goat anti-mouse Cy3 and donkey anti-goat Alexa 488 (1:300, Jackson ImmunoResearch, West Grove, PA).

### Fluorescent Measurements of Intracellular Ca^2+^

DIV 11–15 striatal neurons grown on coverslips were loaded with Ca^2+^ fluorophore Oregon Green 488 BAPTA-1 AM, imaged and quantitated as described [13, 15, 26]. Glutamate (Sigma-Aldrich, St. Louis, MO) was added at various concentrations in the presence of D-(-)-2-Amino-5-phosphonopentanoic acid (APV, NMDA receptor antagonist, 100 μM, Tocris); 6-Cyano-7-nitroquinoxaline-2,3-dione (CNQX, AMPA/Kainate receptor antagonist, 20 μM, Tocris); (2S)-2-Amino-2-[(1S,2S)-2-carboxycycloprop-1-yl]-3-(xanth-9-yl) propanoic acid (LY341495, Group 2/3 mGluR antagonist, 100 nM, Tocris); and 7-(Hydroxyimino)cyclopropa[b]chromen-1a-carboxylate ethyl ester (CPCCOEt, mGluR1 antagonist, 20 μM, Tocris) to detect mGluR5 specific Ca^2+^ responses.

### Fluorescence-Based Ca^2+^ Flux Assay with Microplate Reader

Primary striatal cultures (Div11–15) plated in 96-well plates were loaded with 1 μM Fura-2 AM (Molecular Probes) for 30 min at 37 °C and washed with Hanks’ balanced salt solution (HBSS). The cells were then preincubated with APV (100 μM), CNQX (20 μM), LY341495 (100 nM), CPCCOEt (20 μM), and the impermeable, nontransported mGluR5 antagonist 3-[(1S)-1-amino-1-carboxy-2-(9H-thioxanthen-9-yl)ethyl]cyclobutane-1-carboxylic acid, (LY393053, 20 μM, Eli Lilly, Indianapolis, IN) in HBSS for 20 min at 37 °C prior to measure intracellular mGluR5 specific Ca^2+^ flux. Fura-2 fluorescence was measured using a BioTek™ Synergy™ H4 Hybrid Microplate Reader (BioTek, Winooski, VT). The baseline 340/380 nm excitation ratio for fura-2 was collected for 5 s before injecting various concentration of glutamate. Data were collected for an additional 30 s and then analyzed using Biotek’s Gen5 analysis software. The dose-response curves were fit using the GraphPad Prism 3.0 program (Graphpad Software, San Diego, CA).

### Caged Glutamate Experiments

DIV11–15 striatal cultures were loaded with Oregon green BAPTA-1 AM (Molecular Probes, Eugene, OR) as described [13]. Cells were microinjected with fluoro-ruby (3.2 mg/ml, Molecular Probes) and 20 mM 4-Methoxy-7-nitroindolinyl-caged-l-glutamate (MNI-caged-glutamate, Tocris, Avonmouth, Bristol, United Kingdom) using the single cell electroporator, Axoporator 800A (Molecular Devises, Silicon Valley, CA). Alternatively, MNI-caged glutamate was bath applied to the cells at a concentration of 200 μM. Cells were kept at 37 °C and imaged on an Olympus FluoView™ FV1000 confocal microscope with a SIM scanner. Photo-uncaging was performed using 405 nm laser with Tornado scanning within the region of interest (ROI) for 500 ms. Where indicated, the following antagonists were used at the indicated concentration: APV (100 μM); CNQX (20 μM); LY341495 (100 nM); CPCCOEt (20 μM), LY393053 (20 μM) and 2-Methyl-6-(phenylethynyl)pyridine (MPEP, 10 μM, Tocris). Calcium responses in the ROI and control areas were analyzed using MetaMorph software (Molecular Devises).

## Results

### Glutamate is Sequestered in Neuronal Mitochondria

**Fig. 1:**
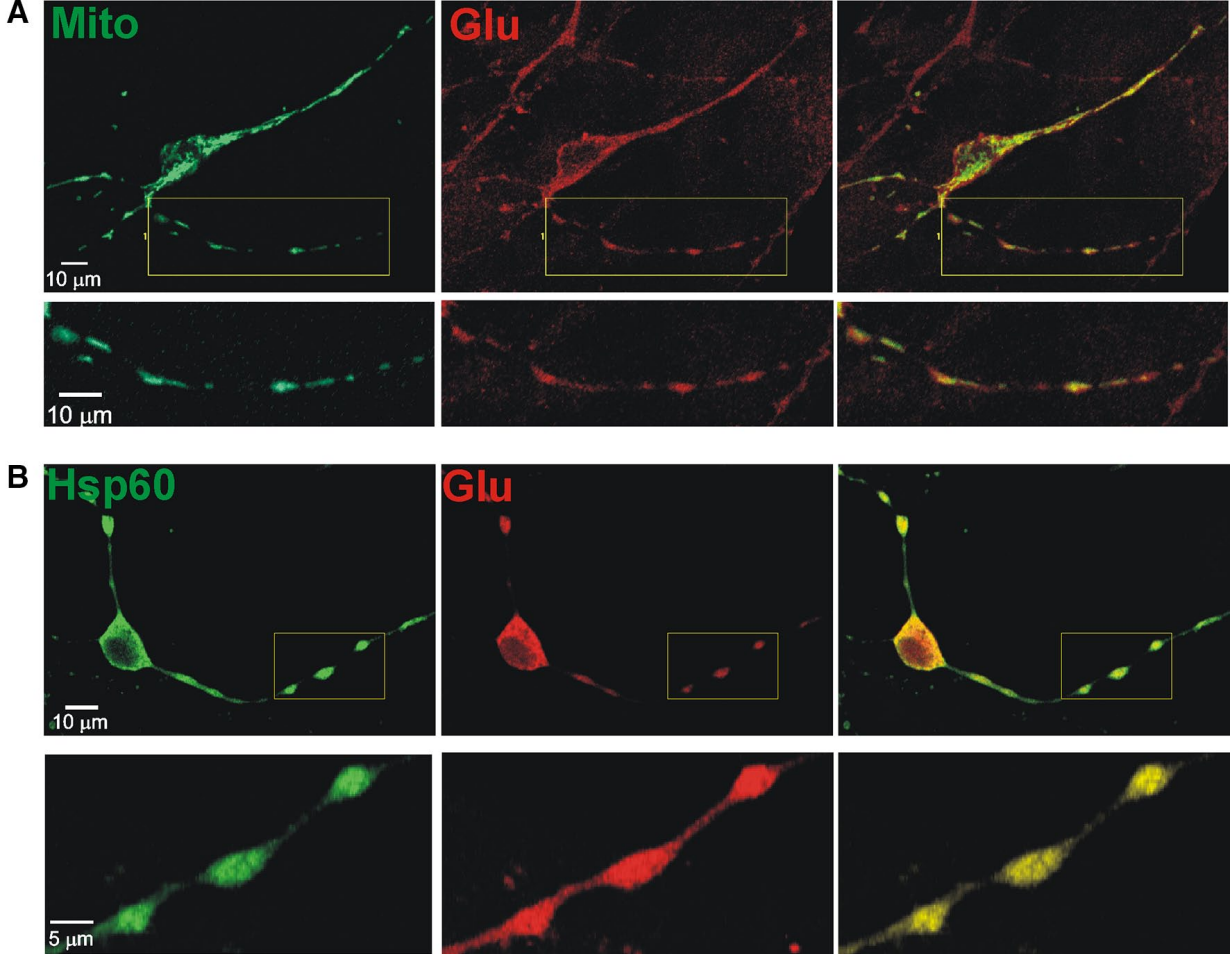
Intracellular glutamate pools are predominantly localized in mitochondria. **a** Glutamate co-localizes with mitochondrial-targeted YFP. Cultured striatal neurons were transfected with mito-YFP (*green*) and stained with anti-glutamate (*red*). **b** Colocalization of glutamate (*red*) with the mitochondrial marker, HSP60 (*green*). For **a, b** magnified images of *boxed area* in *upper panels* are shown below (Color figure online)

Previous studies using techniques such as ^13^C-NMR, ^13^C- and/or ^15^N-GC/MS have provided compelling evidence that glutamate has many fates within the cell. For example, a large proportion of glutamate is taken up by the mitochondria where it is transaminated and serves as a substrate for the TCA cycle [27, 28]. Indeed, anti-glutamate immunogold electron microscopy studies indicate that particles representing glutamate are clustered over mitochondria as well as the nucleus [29]. We have previously shown that mGluR5 is highly expressed in GABA-ergic striatal neurons [13]. To determine whether glutamate could be visualized in striatal neurons, we transfected cultures with mito-YFP to label mitochondria and then fixed and stained for glutamate. In support of EM studies showing anti-glutamate immunogold particles over mitochondria [30], immunofluorescence showed anti-glutamate co-localized with mito-YFP in striatal cell bodies and processes (Fig. 1a). In addition, cultures stained with HSP60, a marker of mitochondria as well as anti-glutamate also showed co-localization (Fig. 1b). Thus the majority of immunoreactive glutamate within these GABA-ergic neurons is compartmentalized in mitochondria.

### “Location” Bias Apparent in Receptor-Mediated Ca^2+^ Responses

**Fig. 2:**
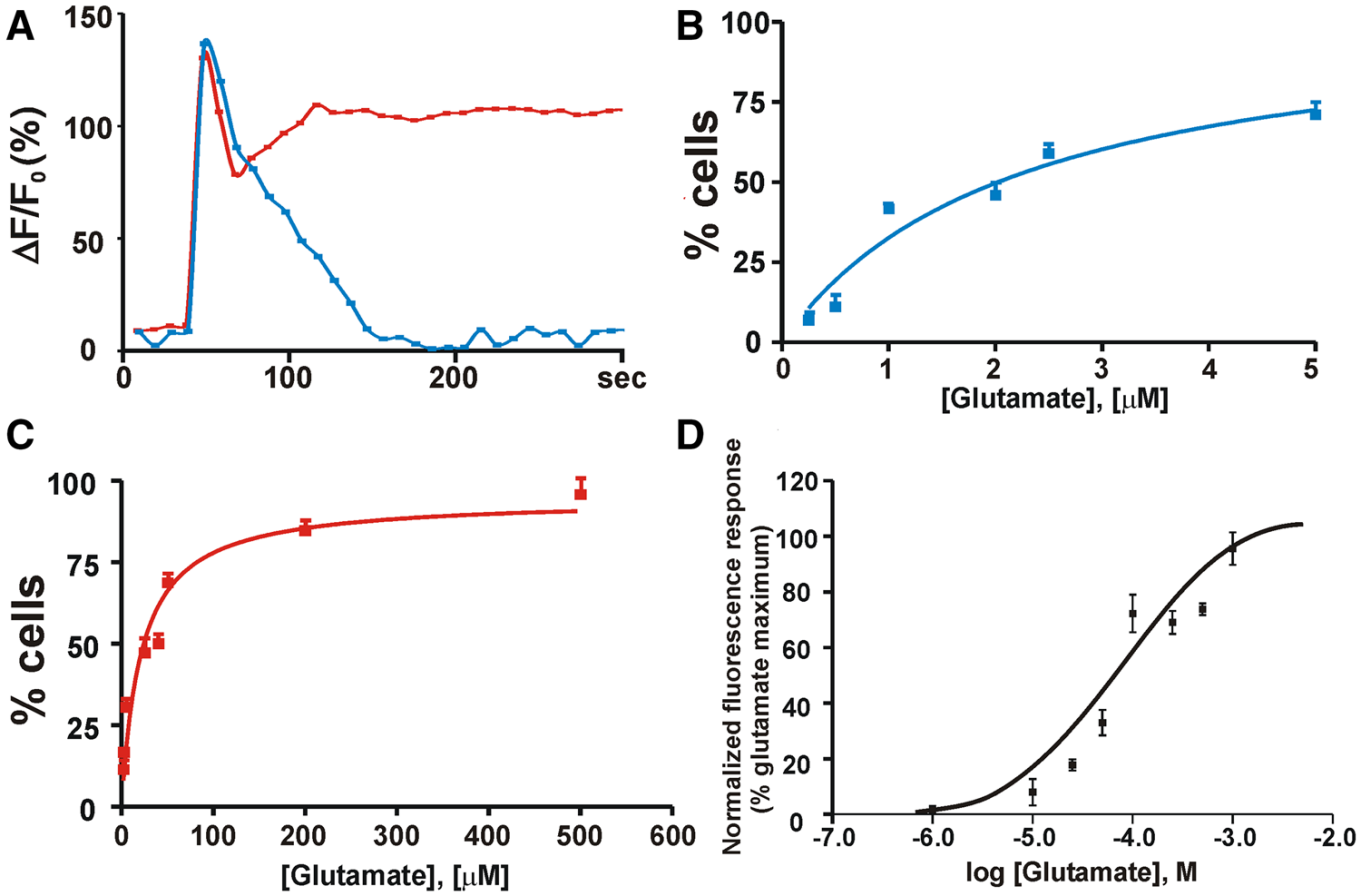
Half-maximal glutamate concentrations associated with intracellular mGluR5-mediated Ca^2+^ responses in striatal neurons. **a**–**c** DIV 11–15 striatal neurons grown on coverslips were loaded with Ca^2+^ fluorophore Oregon Green 488 BAPTA-1 AM and imaged. **a** Glutamate dose-dependency in Ca^2+^ responses; only a single transient peak (*blue*) is observed at glutamate doses below 5 μM whereas both transient and sustained peaks (*red*) are seen with higher glutamate concentrations. **b** The EC_50_ glutamate concentration to stimulate a rapid transient Ca^2+^ response (cell surface) is 2.21 ± 0.8 μM. *Error bars* represent SEM. (N = 3). **c** The EC_50_ glutamate concentration to induce a sustained Ca^2+^ response (intracellular) is 21.4 ± 4.0 μM, *Error bars* represent SEM. (N = 3). **d** DIV 11–15 striatal neurons plated on 96-well plates were loaded with fura-2 AM for Ca^2+^ flux plate reader assay. The baseline 340/380 nm excitation ratio for fura-2 was collected for 5 s before injecting with various concentrations of glutamate. Data were normalized to a glutamate (2 mM) control maximum. Concentration-response curves were generated from the mean data of three experiments. *Error bars* represent SEM. The EC_50_ glutamate concentration for intracellular mGluR5 is 61.3 ± 20.3 μM (Color figure online)

Earlier studies [15] showed no significant differences in glutamate binding at receptors prepared from striatal plasma membrane or intracellular membrane sources. Those studies, however, did not address location-specific receptor responses in terms of function. Therefore, we used real time Ca^2+^ imaging to determine half-maximal glutamate concentrations associated with the plasma membrane or intracellular mGluR5-mediated Ca^2+^ responses. As shown previously [15], glutamate-mediated Ca^2+^ changes consisted of two phases, an initial rapid rise followed by a sustained elevation (Fig. 2a, red trace). Both sets of responses were terminated by the addition of the permeable mGluR5 antagonist, MPEP, whereas cultures pretreated with the impermeable, nontransported antagonist LY393053, only exhibited a sustained Ca^2+^ response pattern (not shown). As shown previously, LY393053 by itself had no effect on Ca^2+^ responses in striatal cultures [13–15]. In contrast, addition of the nontransported agonist, DHPG, led to a rapid transient Ca^2+^ peak (Fig. 2a, blue trace), which could be blocked by LY393053 (not shown). The half-maximal glutamate concentration to stimulate a rapid transient Ca^2+^ response (cell surface) is 2.21 ± 0.8 μM (Fig. 2b) whereas the half-maximal concentration to induce a sustained plateau Ca^2+^ response (intracellular; [15]) is 21.4 ± 4.0 μM (Fig. 2c).

To extend these results, we used a fluorescence-based Ca^2+^ flux plate-reader assay in which cells were loaded with the ratiometric Ca^2+^ indicator Fura-2 AM before Ca^2+^ flux measurement. Previously we used this assay system to show that mGluR5-expressing spinal cord dorsal horn neurons couple to PLC to induce release of Ca^2+^ from intracellular stores [24]. Here, we used this assay to show that the half maximal glutamate concentration for intracellular mGluR5 is 61.3 ± 20.3 μM (Fig. 2d). Presumably, the increased EC_50_ value associated with intracellular mGluR5 reflects properties of the uptake mechanisms involved in glutamate transport into the cell [13, 15]. Collectively, these data show that intracellular, striatal mGluR5 can function independently of signals originating at the cell surface and thus plays a dynamic role in mobilizing Ca^2+^ in a specific, localized manner. In addition these data emphasize that intracellular receptors can be activated with glutamate concentrations far lower than the putative intracellular cytoplasmic concentration, consistent with the notion that glutamate is sequestered in the cell.

### Selective Uncaging of Glutamate Activates Intracellular mGluR5 Within the Cell and in the Dendrites

**Fig. 3:**
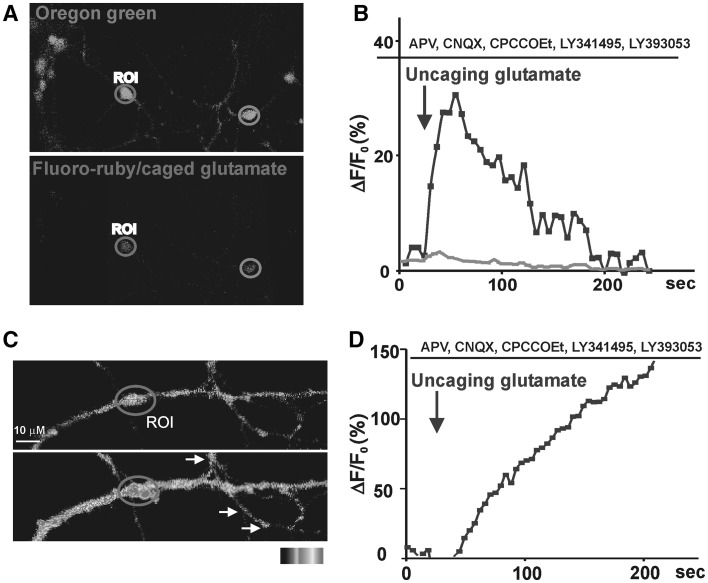
Regionally selective uncaging of glutamate in the presence of cell surface inhibitors activates intracellular mGluR5 in striatal soma (**a, b**) and dendrites (**c, d**). **a, b** Striatal neurons were injected with fluoro-ruby and MNI-caged-glutamate. Uncaging of MNI-glutamate at a somal ROI induced a Ca^2+^ rise at the red ROI whereas no Ca^2+^ changes were seen at the *blue* ROI (N = 5). **c, d** MNI-caged glutamate was bath applied to striatal cultures at a concentration of 200 µM. MNI-glutamate was uncaged on a striatal dendrite at the *red* ROI in the presence of LY53 (20 µM), the NMDA receptor blocker APV (100 µM), the AMPA/Kainate receptor blocker CNQX (20 µM), the mGluR1 blocker CPCCOEt (20 µM) and the Group 2/3 mGluR antagonist LY341495 (100 nM). Uncaged glutamate generated a Ca^2+^ rise at the *red* ROI whereas no Ca^2+^ changes were seen in a different neurite in the same field (*white arrows*) (N = 10) (Color figure online)

To further demonstrate that glutamate activates intracellular mGluR5, we electroporated caged glutamate (MNI-Glu) into individual neurons along with fluoro-ruby to tag recipient cells. Following electroporation, cultures were loaded with Oregon Green BAPTA-1 AM and preincubated with LY393053 as well as ionotropic receptor (NMDA, AMPA, and kainate receptors), mGluR1, and Groups 2 and 3 mGluR antagonists prior to uncaging a ROI (Fig. 3a). Only neuronal somas subjected to laser-induced photolysis (uncaging) showed mGluR5-mediated Ca^2+^ changes (Fig. 3b; red trace), neuronal somas without uncaging showed no change in Ca^2+^ responses (Fig. 3b; blue trace). These data demonstrate that releasing glutamate within the cell can activate intracellular mGluR5.

Because ultrastructure studies have also shown large numbers of mGluR5 gold particles on endoplasmic reticulum (ER) membranes [12], we tested whether mGluR5 expressed on dendritic ER or endosomal membranes can mediate local Ca^2+^ rises. To do so intracellular receptors were pharmacologically isolated by blocking cell surface mGluR5 with LY393053 as well as mGluR1, ionotropic and Group 2/3 mGluR targets. In addition to the antagonists, the extracellular buffer also contained MNI-glutamate which was uncaged in an ROI on a striatal dendrite at least 20 μm away from the cell body (Fig. 3c). Only the region of the dendrite juxtaposed to the uncaging spot exhibited a change in fluorescence whereas the surrounding regions did not (Fig. 3c, d). MPEP blocked all Ca^2+^ responses (not shown). These data emphasize the notion that some of the signaling originates in the ER, an organelle not easily assayed other than using the selective tools described here. Thus akin to results in the hippocampus [23], our findings indicate that activation of dendritic, intracellular mGluR5 also leads to in situ Ca^2+^ changes with neither input to nor output from the cell soma.

## Discussion

In addition to functioning as a neurotransmitter, glutamate serves many metabolic roles within the cell such as acting as a building block for protein synthesis, playing a role in energy metabolism, and transferring reducing equivalents from the cytoplasm to the mitochondria [31]. Given these myriad tasks, it is not surprising that intracellular glutamate concentrations are thought to be in the mM range. Contrary to this notion, here we show that glutamate is largely compartmentalized in mitochondria, at least in striatal neurons. Moreover, the EC_50_ for glutamate activation of intracellular mGluR5 is ~61 μM, a value inconsistent with high concentrations of “free” glutamate within the cytoplasm. Finally uncaging glutamate within the neuronal soma led to a rapid mGluR5-mediated Ca^2+^ response further demonstrating intracellular glutamate activation of intracellular receptors.

These studies do not rule out the possibility that glutamate could also be generated in situ. For example, glutamate may also be generated within the nucleus by phosphate-activated l-type glutaminase [32]. Studies by Aledo and co-workers have shown that throughout the brain of humans, monkeys, rats and many other species, l-type glutaminase can be found primarily in the nucleus where it exhibits kinetics consistent with those of the better characterized liver enzyme [32]. More recently, GIP, a scaffolding protein known to bind to a PDZ domain within l-type glutaminase, has been shown to be associated with ER and nuclear membranes [33]. Thus, in addition to transport from extracellular sources and in situ cytoplasmic production, enzymes and scaffolding proteins exist to localize glutamate production near intracellular mGluR5.

In conclusion, uncertainties regarding the concentrations, fate and function of intracellular glutamate as well as findings showing the highly compartmentalized nature of glutamate production and disposition have obscured studies on intracellular receptors and transporters such as mGluR1 [22], mGluR5, and EAAT3. These uncertainties have delayed progress in discerning the role of intracellular glutamate as well as the functional consequences of its production and/or uptake. For instance, the presence of EAAT3 on postsynaptic processes as well as intracellular membranes suggests that glutamate could be targeted to other receptors/channels that might also be present on intracellular membranes. Because these transporters work in “reverse”, glutamate could bind to any ligand binding domain present on the same membrane as a given transporter. Given that such proteins would still maintain their “signaling” domains within the cytoplasm, entirely new signaling molecules might be associated with these intracellular receptors. Akin to β-arrestin-scaffolded GPCRs, one set of second messenger systems might be exchanged for another [34]. In support of this notion, our data show that activated intracellular receptors generate sustained Ca^2+^ responses and trigger completely different signaling pathways [15]. As mGluR5 plays important roles in development, synaptic function, and learning and memory as well as pathological roles in Fragile X syndrome, anxiety, addiction, and Parkinson disease, understanding the long term consequences of intracellular receptor activation might lead to novel therapeutic strategies for these disorders. It seems likely that besides receptor-specific characteristics and signaling partners that the many factors that modulate glutamate metabolism such as transporter distribution, fluctuation of metabolite concentration, and regulation of the many enzymes involved in its production will also play an important role in receptor activation.
